# Effect of *Zataria multiflora* essential oil and potassium sorbate on inoculated *Listeria monocytogenes*, microbial and chemical quality of raw trout fillet during refrigerator storage

**DOI:** 10.1002/fsn3.2259

**Published:** 2021-03-26

**Authors:** Forough Motavaf, Alireza Mirvaghefi, Hamid Farahmand, Seyed Vali Hosseini

**Affiliations:** ^1^ Department of Fisheries Faculty of Natural Resources University of Tehran Karaj Iran

**Keywords:** *Listeria monocytogenes*, potassium sorbate, rainbow trout fillet, shelf life, *Zataria multiflora*

## Abstract

Human listeriosis is predominantly associated with contaminated food consumption, including seafood, shrimp, and RTE foods. *Listeria monocytogenes* is a foodborne pathogen that is mainly found in freshwater, seawater, and fish mucus. Seafood contamination can occur during food processing. *L.monocytogenes* levels of below 100 cfu/g can be found in seafood samples. The present study was conducted to investigates the effect of Zataria multiflora essential oil (ZEO) and potassium sorbate (PS) on microbial and chemical changes in raw rainbow trout at 4°C to extent shelf life and improve food safety. First, the chemical compositions of ZEO were identified. Then, different percentage of ZEO (1.5, 0.8, and 0.5%) and PS (2%) were inoculated in raw fish fillets and analyzed for TVC, TBA, TVB‐N, pH, sensory attributes, *Pseudomonas aeruginosa,* and inoculated *L. monocytogenes* (1 × 10^5^ cfu/g) survival at 4°C for 12 days. The best sensory evaluation score was observed for the samples treated with 0.8% and 1.5% ZEO. Overall, this study results indicated that the treatment of rainbow trout fillet with 1.5% ZEO is the best method for controlling the growth of *L. monocytogenes* at refrigerator temperature without any undesirable sensory effects.

## Graphical abstract

In this study, the antibacterial effect of Zataria multiflora essential oil and potassium sorbate on Listeria monocytogenes in Rainbow trout fillet was investigated. Based on the results of chemical, microbial, and sensory evaluation tests, 1.5% Zataria multiflora essential oil preserved Rainbow Trout fillet with the best quality for 12 days.

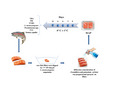

## 1 INTRODUCTION

Foodborne infections are one of the most expensive and serious public health concerns in the world. With a 30% mortality rate, *L. monocytogenes* is an important foodborne pathogen (Botsaris et al., 2016). *L. monocytogenes* is a gram‐positive bacterium that invades cells as an internal parasite (McMullen & Freitag, 2015). This species is an aerobic to facultative anaerobic bacillus and can survive on the surface of food processing equipment and seafood like marinated or cold‐smoked fishes by forming a biofilm (Papaioannou et al., 2018). In the seafood processing industry, the transmission of *L. monocytogenes* from raw materials can contaminate the final products such as cold‐smoked. Similarly, strains of *L. monocytogenes* that are already present in the environment can also contaminate final products (Aymerich et al., 2019).

*L. monocytogenes* previously found in catfish and final raw products made from catfish (Dhowlaghar et al., 2017). In a study on fresh rainbow trout in the United States, 54% of samples on the retail market tested positive for *L. monocytogenes*. Another study on raw rainbow trout sold in Iran's retail stores reported a contamination rate of 12.5% (Abdollahzadeh et al., 2016).

Several of chemical compounds can control and eliminate this pathogen, such as potassium sorbate (PS) and sodium benzoate (Necidová et al., 2019; Das et al., 2016). Because chemical preservatives are carcinogenic and toxic, researchers have suggested that these compounds be replaced with natural preservatives (Cui et al., 2020; Guitián et al., 2019). Natural plant extracts and spices have long been used as seasoning for meats. Plant extracts also exhibit antibacterial activity by destabilizing the bacterial plasma membrane and disrupting enzyme activity and genetic material in bacteria (Burt, 2004; Pilevar et al., 2020).

*Zataria multiflora* (*Z. multiflora*) is a species in the Laminaceae family that exclusively grows in Afghanistan, Iran, and Pakistan. In Iran, this plant is known as “Aavishan‐e Shirazi” (Shirazi thyme) and is used in traditional medicine as an antiseptic, analgesic, and antispasmodic (Mahmoudvand et al., 2017). In local cuisine, *Z. multiflora* is used as a seasoning in a wide range of dishes. The main components of *Z. multiflora* essential oil (ZEO) are phenolic compounds such as carvacrol, thymol, γ‐terpienene, and P‐cymene (Niczad et al., 2019). The structure of carvacrol and thymol is very similar. Both of them damage the cell wall of gram‐positive bacteria by permeating the plasma membrane (Burt, 2004). Studies show the anti‐listeria and antioxidant activity of ZEO in several foods such as seafood, ham, cooked beef, chicken, and vegetables (Abbasi et al., 2020; Desai et al., 2012; Raeisi et al., 2016).

These properties have attracted considerable attention to ZEO in the food industry researchers. However, using essential oils as food antibacterial agents might change the taste, smell, and organoleptic properties undesirably. Generally, adding essential oils to food is limited due to the unwanted taste in the final product. Therefore, it is recommended to use essential oils in concert with other antibacterial compounds and other preservation techniques such as mild heat, hydrostatic pressure, and adding sodium citrate and monolaurin (Calo et al., 2015).

Multiple factors, such as species, fish size, temperature, physical conditions, harvest conditions, and transportation, affect the shelf life of fish (Dawson et al., 2018). In this study, rainbow trout was selected as the substrate to investigate the antibacterial and antioxidant properties of ZEO against *L. monocytogenes*. Rainbow trout fillets were selected due to the widespread farming and availability of different species in Iran.

## 2 MATERIAL AND METHODS

### 2.1 ZEO

ZEO (Code 706 013, 40 grams jar) purchased from Barij Essence Pharmaceutical Company (Kashan, Iran), and it was stored in the dark bottle at refrigerator temperature (4°C ± 1°C).

### 2.2 Chemical composition of ZEO

The essential oil was analyzed by gas chromatography (GC) (Thermoquest 2000, UK) equipped with DB5 capillary column (30 × 0.25mm ID × 0.25 µm film thickness). Data obtained under the following analytical conditions: initial temperature 50°C; program rate 2.5°C; final temperature 265°C; and injector temperature 250°C. Helium was used as a carrier gas, and the split ratio was 120. In addition, essential oil analysis was performed using gas chromatography‐mass spectrometry (GC–MS) (Termoques Finningan, UK) and similar conditions mentioned above. The MS ran under the electron ionization mode at the ionization energy of 70eV (Adams, 1989).

### 2.3 Preparation of* *ZEO oil and PS

ZEO (Code 706 013, 40 grams jar) purchased from Barij Essence Pharmaceutical Company (Kashan, Iran). 0.5%, 0.8%, and 1.5% (w/w) solutions of Z. *multiflora *in glycerin 1.5% (w/v) (Merck, Germany) were prepared (Hashemi et al., 2020). Glycerin was used to improve solubility. Glycerin works as an emulsifier for blending oil‐based ingredients such as essential oils. Solutions were prepared with sterile distilled water. Each concentration of essential oil was prepared with 500 ml of sterile distilled water and glycerin mixture. To prepare 2% (w/v) of PS (Merck, Germany) solution, the appropriate amount of potassium sorbate was dissolved in distilled water. This percentage of potassium sorbate is safe in the food industry and is not harmful to the consumer (Das et al., 2016).

### 2.4 Bacterial strain

The standard strain of *L. monocytogenes* PTCC 1,163 was obtained from the Razi Vaccine and Serum Research Institute (Persian Type Culture Collection (PTCC), Tehran, Iran). The strain was then reactivated in glycerol stock containing 20% (w/v) glycerol in Brain Heart Infusion (BHI) broth (Scharlua, Spain) and stored at −20°C. Subsequently, the isolated strain was inoculated into vials containing 15 ml BHI and incubated at 37°C at 150 rpm for 24 hr, with two consecutive transfers (Ojagh et al., 2010). The output was then centrifuged three times at 6,000 rpm for 5 min to separate bacterial cells from BHI. After twice centrifuging, the supernatant was removed, followed by twice washing, resuspended in PBS (50 mM K2HPO4/KH2PO4; pH 7.4; Sigma‐Aldrich, St. Louis, USA) to obtain standard cell suspensions. For bacteria counting, the optical density (OD) method at 600 nm (OD600) was used by a spectrophotometer UV‐1204 (Shimadzu, Kyoto, Japan) (according to the pretest, 0.1–0.08 OD was equal to 1 × 108 cfu/g). After preparing serial dilution, the raw fish fillets were inoculated by 1 × 105 cfu/g of *L. monocytogenes* (de Sousa Guedes & de Souza, 2018).

### 2.5 Inoculated rainbow trout fillets

15 kg of rainbow trout was purchased from Alborz Aquatic Plant, immediately placed in iceboxes, and transferred to the Aquatic Processing Laboratory of the Faculty of Fisheries, University of Tehran. The fish were gutted, bled, and cut into 200 grams fillets. All fillets were then immersed in a suspension of bacteria for 5 min, except for the control treatments. Then, water and the solution containing bacteria were allowed to drip out. 500 ml of each ZEO solution (0.5%, 0.8%, and 1.5%) and PS solution 2% were prepared and sprayed on fillets. After 5 min, all stained fillets were transported to the packaging laboratory of the University of Tehran Food Science and Industry faculty for packaging. The fillets were treated with 6 treatments: control treatment (no bacteria or antibacterial agents), ZEO solution (0.5, 0.8, and 1.5%), PS solution 2%, and the last treatment, which included pure bacterial stain (*L. monocytogenes*). Chemical, microbial, and sensory evaluation indexes were measured on days 0, 3, 6, 9, and 12. All treatments were aseptically packed by a modified atmosphere packaging machine with 30% N2, 30% O2, and 40% CO2 and transferred to another laboratory then stored at 4°C ± 1°C (Goulas & Kontominas, 2007).

### 2.6 Microbial tests

For each treatment, five packages of 200 grams of meat were used. 10 grams of each sample was blended with 90 ml of normal saline solution (NSS) by stomacher blender (Seward Stomacher 80) at normal speed for 60 s. Then, 10‐fold serial dilutions were prepared. Total viable count (TVC) was estimated by standard plate count technique. Plate count agar (PCA) was used for the total viable count (TVC) after incubation at 37°C for 24 hr, and the number of bacteria was expressed as log cfu/g. All treatments had two replications (Shokri et al., 2015). *P*. *aeruginosa* were counted using *P*. *aeruginosa* Cetrimide agar (Laboratorios Conda S.A.) after 48 hr of incubation at 37°C. Selective media are used for the growth of only selected microorganisms (Shokri et al., 2015). *L. monocytogenes* population were detected by seeding 0.1 ml of serial dilutions on CHROMagar^TM^ Listeria agar (CHROMagar Microbiology, France). Subsequently, the plates were incubated at 37°C for 48 hr. CHROMagar helps to easily differentiate Listeria monocytogenes from other Listeria directly at the isolation step: The colonies were blue and surrounded by a white halo due to a specific phospholipase activity. The number of Listeria per gram of fillet was expressed as log cfu/g (Hegde et al., 2007).

### 2.7 Chemical tests

#### 2.7.1 Determination of thiobarbituric acid (TBA)

Lipid oxidation is one of the major causes of quality deterioration in meat. Several methods have been developed to assess lipid oxidation products in raw foods. The thiobarbituric acid (TBA) test is most widely used to quantify lipid oxidation products in meat products because it is fast and straightforward. The TBA test determines the amount of malondialdehyde (MDA), a major secondary by product of lipid oxidation, in a sample (Zeb & Ullah, 2016). First, 2 grams samples blended with 8 ml of 4% perchloric acid and 1 ml BHA solution (to prevent lipid oxidation during analysis). Then, samples were homogenized by a stomacher blender (Seward Stomacher 80), finally placed in a dark cabinet for 30 min. After this phase, samples were centrifuged. Then, 5 ml of the filtered supernatant and 5 ml of 0.03% solution of thiobarbituric acid was transferred to the test tube. The mixture was placed in 95°C water for 30 min. After cooling down the tubes at ambient temperature, the absorbance solution was read for each spectrum by a UV‐2100 spectrophotometer (Unico, USA) at 530 nm and malondialdehyde concentration (mg/kg) (Chatzikyriakidou & Katsanidis, 2012).

#### 2.7.2 Determination of Total Volatile Basic Nitrogen (TVB‐N)

TVB‐N refers to a wide range of basic volatile compounds such as ammonia, methylamine, dimethylamine, trimethylamine, and other similar compounds produced during microbial activity, which are used as indicators for meat decomposition. The sign of decomposition threshold in rainbow trout muscle is 25 mg TVB‐N per 100 grams of meat (Arashisar et al., 2004). First, 2 grams of the sample was mixed with 8 ml of 4% trichloroacetic acid (TCA) solution and homogenized by a stomacher blender (Seward Stomacher 80). The homogenized solution was then centrifuged for 30 min at 4,000 rpm. The supernatant was poured into the outer ring of the Conway apparatus. Subsequently, 1% boric acid and a mixed bromocresol green and methyl red were added to the inner ring. Then, potassium carbonate (Merck, Germany) was added to the outer ring to start the reaction. The final output was incubated at 37 ˚C for an hour. Subsequently, the inner ring solution was titrated with 0.02 N HCl until the color of the solution changed to pink. (Rawdkuen et al., 2010):

TVB‐*N* (mg N/100 g sample) = {14 (N) (A‐B) (V) (100)}/ M.

In the above formula, N is the normal value of the chloride acid used to titrate the samples. A and B, respectively, are the volume (ml) of acid used for titration of the sample and the volume (ml) of acid used to titrate the control sample. V is the volume (ml) of the phase sample liquid after centrifugation, and M is the initial sample weight in grams.

#### 2.7.3 Determination of pH

5 g of the fish filet was homogenized, mixed with 45 ml of distilled water, and placed on a shaker with 180 rpm (Model G24 Environmental shaker, New Brunswick Scientific Co., NJ, USA). The pH of the sample was analyzed by Corning pH meter (Model 430, Corning Inc., NY, USA) (Suvanich et al., 2000).

### 2.8 Sensory evaluation

For sensory evaluation, the noninoculated fish fillet samples were sliced into 200 g pieces. To prepare treatments, each sample sprayed with ZEO solution (0.5%, 0.8%, or 1.5%) or PS solution 2% solution. Each sample was wrapped in aluminum foil individually and cooked in a steam cooker (MultiGourmet FS20, Braun, Germany) for 30 min. Ten university students were chosen and trained according to the American Meat Science Association (AMSA) (,^2016^, February 11) guidelines to evaluate the sensory property of fish filets treated with ZEO including, color, odor, taste, texture, and overall acceptability attributes. The panelists graded test sheets from excellent (9), very good (8), good (7), acceptable (6), to the poor (5). A sensory score of less than 5 was considered unacceptable. Furthermore, at the end of the test, panelists scored the samples based on overall customer satisfaction from 1 to 9.

### 2.9 Statistical Analysis

The obtained data were statistically analyzed by one‐way ANOVA using the SPSS software, version 21 (IBM software, NY, 201 USA). Duncan test and one‐way analysis of variance (ANOVA) were used at 0.05 levels to examine the significant difference between treatments.

## 3 RESULTS AND DISCUSSION

### 3.1 Chemical composition of ZEO

Table 1 shows the ratio of essential oil components based on GC and GC‐MS. As can be seen, carvacrol is the chief component of essential oil (66.2%), followed by thymol (26.5%) and ρ‐cymene (3.98%). Some differences were observed between our findings and Mahmoudvand et al., (2017) results. They reported thymol (40.8%), carvacrol (27.8%), ρ‐cymene (8.4%), γ‐terpinene (4%), and linalool (0.78%) as the most representative constituents of ZEO. However, our results agreed with Barkhori‐Mehni et al., (2017) that reported carvacrol (51.55%), thymol (25.49%), and p‐cymene (5.23%) were the most representative components of ZEO. According to Shahbazi et al., (2016), the chemical composition variation is mainly due to the differences in herbal species, age, ecotypes, and other environmental factors.

**TABLE 1:** Essential oil composition of *Z. multiflora* identified by gas chromatography and gas chromatography–mass spectrometry

Compound	Retention index	Percentage
Carvacrol	1,300	66.2
Thujene	930	0.12
Thymol	1,342	26.5
Linalool	1,086	0.78
P‐cymene	1,015	3.98
Eucalyptol	1,024	0.31
Trans‐Caryophyllene	1,418	0.41
Alpha‐pinene	937	1.02
Beta‐pinene	976	0.4
Sum		99.72

### 3.2 Survival of *L. monocytogenes*

**FIGURE 1:**
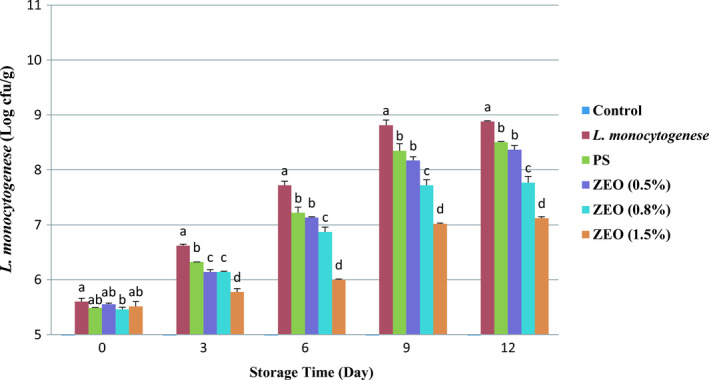
Average number (± *SD*) of *L*. *monocytogenes* in rainbow trout fillets during 12 days storage at 4°C. Different letters indicate significant differences between days of storage

Different treatments significantly affected the number of *L. monocytogenes,* and meaningful differences were observed between different treatments (*p* <.05, *p* <.001). The number of bacteria in the control treatment was zero (Figure 1). Storage time also had a meaningful effect on the bacteria survival (*p* <.001), and significant changes were observed in the number of bacteria during 12 days of storage. The most inhibitory effect on L. *monocytogenes* growth was seen in treatment with 1.5% ZEO on day 3 (6 ± 0.06 log cfu/g), which agreed with a previous study, Yaghoubzadeh and Safari (2015), who reported, 1.2% ZEO had the best result in controlling *L.monocytogenes* inoculated into ground silver carp (Hypophthalmichthys molitrix). The relationship between the main factors of the test, treatments, and storage time was significant. In the current study, the number of *L. monocytogenes* for rainbow trout fillets ranged from 5.51 ± 0.08 log cfu/g in treatment with 1.5% ZEO to 9.79 ± 0.14 log cfu/g in the *L. monocytogenes* treatment. In different ZEO treatments, the lowest number of Listeria bacteria was observed in the 1.5% ZEO from 5.51 ± 0.08 log cfu/g to 7.12 ± 0.02 log cfu/g (Figure 1). 1.5% ZEO treatment showed the best anti‐listeria effect. Pilevar et al., (2020) and Tajik et al., (2015) found similar inhibition effects of ZEO on growth *L.monocytogenes* in broth and minced rainbow trout and raw buffalo patty, respectively. The high anti‐listeria power of ZEO is associated with the large ratio of phenolic compounds. As table 1 data show, the main phenolic compounds in the ZEO were carvacrol (66.2%) and thymol (26.5%). The hydrophobic nature of phenolic compounds in essential oils dissolves the hydrophobic region of the bacterial cytoplasmic membrane, and ruptures the external layer of the membrane, therefore inhibiting bacterial growth (Niczad et al., 2019). After 30 min of contact, carvacrol destroys the L. monocytogenes cell membrane more efficiently. 150–200 ppm of pure carvacrol or thymol significantly inhibits the growth of *L. monocytogenes* (Arioli et al., 2019).

### 3.3 Evaluation of *P. aeruginosa*

**FIGURE 2:**
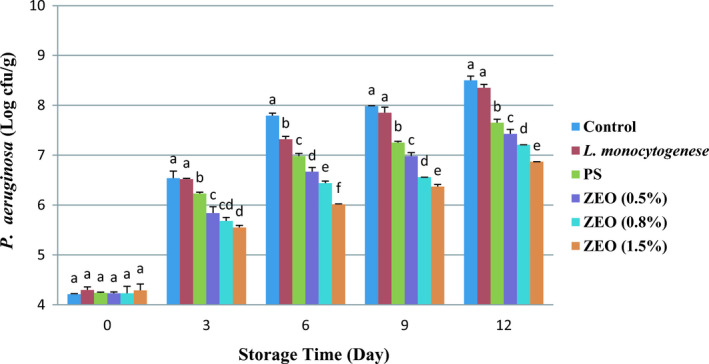
Average number (± *SD*) of *P*. *aeruginosa* bacteria in rainbow trout fillets during 12 days storage at 4°C. Different letters indicate significant differences between days of storage

*P. aeruginosa* is a gram‐negative, rod‐shaped bacterium and one of the most important psychrophilic bacteria that can decompose fish products during cold storage. *P. aeruginosa* induced quality changes such as sensory, physiochemical, and microbiological in the shelf life of rainbow trout fillets during refrigerator storage (Kamani et al., 2020). Results of this study indicated the significant effect of different treatments (*p* <.001), different days of storage (*p* <.001), and the relationship between these two factors (*p* <.001) on the numbers of *P. aeruginosa*. These results showed the initial population of *P. aeruginosa* was ranged from 4.29 ± 0.12 log cfu/g in the treatments with 1.5% ZEO to 8.5 ± 0.08 log cfu/g in the control treatment. The number of *P. aeruginosa* was almost similar in the zero‐day sampling for all treatments and increased after 3 days. On day 12, the highest amount of *P. aeruginosa* was observed in the control sample (8.5 ± 0.08 log cfu/g) (Figure 2). The number of *P. aeruginosa* reached the highest in all treatments on day 12. The lowest number of *P. aeruginosa* in different treatments was present in 1.5% ZEO. The phenolic compounds in essential oil cause this reduction growth of *P. aeruginosa* in 1.5% ZEO. These results agreed with the previous study Barkhori‐Mehni et al., (2017) on the antibacterial activity of ZEO against some fish spoilage bacteria. Their findings showed that *P. aeruginosa* is the most resistant bacterium among *Aeromonas hydrophila*, *Pseudomonas fluorescens*, *Shewanella putrefaciens*, *Bacillus subtilis*, and *Escherichia coli* to the antibacterial properties of ZEO. This result indicates the effect of the antibacterial substance of ZEO. *P. aeruginosa* had faster and dominant growth than other bacteria in all treatments and in different days of storage, which is in line with Heydari‐Majd et al., (2019) findings. They studied the new active nanocomposite film‐based on ZEO for the preservation of refrigerated Otolithes ruber fillets. Their results showed that 1.5% ZEO had the most inhibition on *P. aeruginosa *growth.

### 3.4 Total Viable Count (TVC)

**FIGURE 3:**
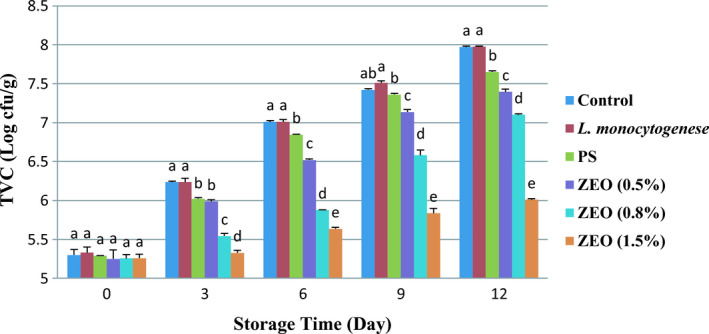
Average number (± *SD*) of TVC in rainbow trout fillets at different during 12 days storage at 4°C. Different letters indicate significant differences between days of storage

Figure 3 shows the total mesophilic aerobic bacteria level significantly differed between all treatments on different days of storage (*p* <.05). The relation between the treatments and storage time also significantly affected the bacteria level in the samples (*p* <.05). The highest bacterial level in the control treatment and the pure bacteria (*L. monocytogenes*) treatment was observed on day 12. During the storage period, the lowest bacteria level was observed in the 1.5% ZEO. These agreed with Khedri and Roomiani (2019) results, which studied the effects of ZEO on silver carp fillets and reported that 1.5% of ZEO nanoemulsion had a meaningful impact on decreasing TVC. In the current study, initial TVC for rainbow trout fillets ranged from 5.25 ± 0.05 log cfu/g in 1.5% ZEO treatment to 7.97 ± 0.01 log cfu/g the control group (*p* <.05). According to other studies on freshwater fish (e.g., tilapia, bass, rainbow trout, and silver perch), TVC depends on water and ambient temperature, and the bacterial load of freshwater fish was about 10^2^–10^6^ (cfu/g) (Chytiri et al., 2004). On day 12, the lowest TVC was observed in 1.5% ZEO treatment. Similar results were reported in another study on nanoemulsions containing ZEO on microbial quality of trout fillet (Khanzadi et al., 2020). The TVC standard limit in rainbow trout fillet is 6 log cfu/g. In this study, the TVC amount in 1.5% ZEO treatment reached its maximum limitation on day 12. The 1.5% ZEO decreased the bacterial load because of its antibacterial compounds includes carvacrol and thymol. This type of compound can have synergistic effects. Carvacrol and thymol are structurally very similar, and both have a hydroxyl group at different locations on their phenolic ring (Burt, 2004). The hydroxyl group of carvacrol, thymol, and the presence of a delocalized electron system (double bonds) empowers carvacrol and thymol to perform as a proton exchanger, thus the gradient reduction across the cytoplasmic membrane, consequently resulting in a collapse of the proton motive force and depletion of the ATP pool subsequently leading to cell death (Arioli et al., 2019; Burt, 2004; Kachur & Suntres, 2020). Indeed, after exposure to thymol and carvacrol, increased membrane permeability is a sign of cell membrane damage by terpenes, acting as substitutional impurities in the phospholipidic bilayer (Cristani et al., 2007). This study data acknowledged the membrane permeabilization due to terpene activity, already reported for gram‐positive and gram‐negative bacteria (Kachur & Suntres, 2020; Memar et al., 2017; Sharifi‐Rad et al., 2017).

### 3.5 Thiobarbituric Acid Index (TBA)

**FIGURE 4:**
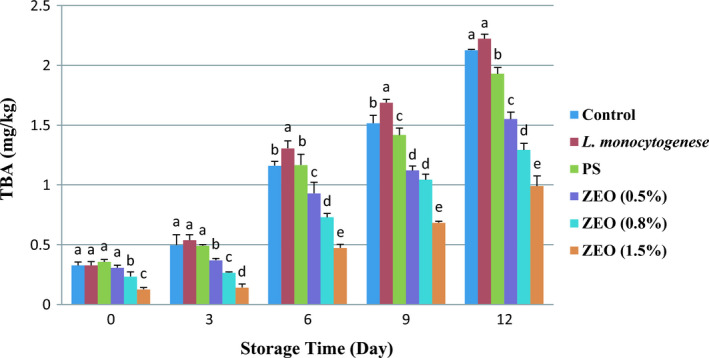
Mean values (± *SD*) of TBA in rainbow trout fillets at different during 12 days storage at 4°C. Different letters indicate significant differences between days of storage

Thiobarbituric acid is a secondary product of fat oxidation. It is extensively used as an indicator for fat oxidation evaluation (Raeisi et al., 2015). Figure 4 shows thiobarbituric acid levels increased during storage time in the refrigerator in all treatments. The trend of TBA in different treatments shows that the pure *L. monocytogenes* treatment had the highest values of TBA from 0.53 ± 0.04 mg/kg to 2.22 ± 0.03 mg/kg on days 3, 6, 9, and 12 (Figure 4). ANOVA results indicated the significant effect of different ZEO treatments on TBA (*p* <.001). Storage time also significantly affected TBA level in different treatments (*p* <.05, *p* <.001). The pure *L. monocytogenes* and control treatment had high TBA levels during the storage period, which was significantly higher than ZEO treatments (Figure 4). These results align with Lashkari et al., (2020) studied on shelf life extension of veal meat by ZEO, which reported that 1.5% of ZEO had the greatest impact on decreasing TBA. The TBA acceptable level for fish meat consumers is about 1–2 mg of malondialdehyde per kg. There is TBA limitation due to unpleasant odor and taste (Khedri & Roomiani, 2019). In this study, the acceptable TBA level reached its limitation in control (1.16 ± 0.03 mg/kg), pure *L. monocytogenes* (1.3 ± 0.06 mg/kg), and PS (1.41 ± 0.06 mg/kg) treatments on day 6. 0.5% and 0.8% of ZEO treatments around day 9, and 1.5% of ZEO treatment on day 12 reached this limitation. TBA changed in 1.5% ZEO treatment from 0.12 ± 0.01 mg/kg on day 0 to 0.98 ± 0.08 mg/kg on day 12. These indicate that ZEO prevents the formation of free radicals due to the presence of phenolic antioxidants (carvacrol and thymol), thus delays the fat oxidation. In 2020, Lashkari et al. reported phenolic compounds in essential oils reduce oxidative strength. Carvacrol and thymol are two main phenolic compounds in ZEO. Hence, it could be concluded that ZEO has relatively significant anti‐radical activity. Several studies reported the key role of phenolic compounds such as carvacrol and thymol in the neutralization of free radicals (Khedri & Roomiani, 2019; Lashkari et al., 2020; Raeisi et al., 2015).

### 3.6 Total Volatile Basic Nitrogen (TVB‐N)

**FIGURE 5:**
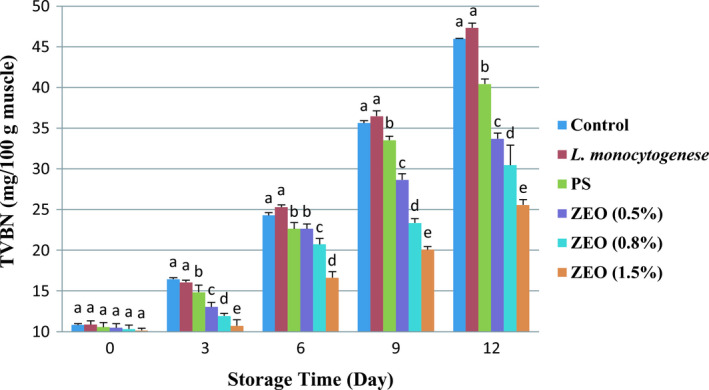
Mean values (± *SD*) for TVB‐N in rainbow trout fillets at different during 12 days storage at 4°C. Different letters indicate significant differences between days of storage

Volatile nitrogenous bases are mainly produced through bacterial decomposition. Various compounds, including ammonia, methylamine, dimethylamine, and trimethylamine, contribute to TVB‐N. These compounds are derived from the activity of bacteria responsible for the degradation and production of internal enzymes (Khedri & Roomiani, 2019; Raeisi et al., 2015). Nitrogenous compounds are often used to indicate the decomposition of fish (Raeisi et al., 2015). According to the ANOVA results, different treatments (*p* <.05), days of storage (*p* <.001), and the relation between these two factors (*p* <.001) significantly affected TVB‐*N* (*p* <.05). Figure 5 shows TVB‐N levels of different treatments were the same on the zero‐day of storage but increased over time in all treatments. Over storage time, the amount of volatile nitrogen increased. The increase in TVB‐N level was slow in the first days but intensified on day 6. The highest amount of TVB‐N was observed in pure *L. monocytogenes* treatment from 10.87 ± 0.44 mg/100g muscle to 47.31 ± 0.58 mg/100g muscle on day 12 due to the more significant number of bacteria. In the early days, bacterial growth is slow (Lag Phase); then, the growth rate increased. TVB‐N production in food causes an unpleasant odor similar to spoilage. The maximum acceptable amount of TVB‐N for rainbow trout is 25 mg per 100 grams of the fillet (Arashisar et al., 2004). According to this study, the lowest TVB‐N rate was recorded for 1.5% ZEO (10.14 ± 0.25 mg/100g muscle). In 1.5% ZEO treatment, TVB‐N level was acceptable until day 9 (20.06 ± 0.38 mg/100g muscle), but this amount was a little too much on day 12 (25.55 ± 0.65 mg/100g muscle). These results agreed with the previous study on the effect of ZEO physicochemical properties of rainbow trout fillets during cold storage (Shadman et al., 2017), which used 0.5% and 1% of ZEO and found 1% of ZEO had the best result in decreasing TVB‐N amount. These outcomes showed that ZEO effectively reduces volatile nitrogen bases, attributed to the higher carvacrol and thymol levels in these treatments, which is in line with Raeisi et al., (2015) study on ZEO and grape seed oil on the shelf life of rainbow trout fillets. They found the minimum level of TVB‐N was measured in the fillets coated with a high percentage of ZEO.

### 3.7 pH

**FIGURE 6:**
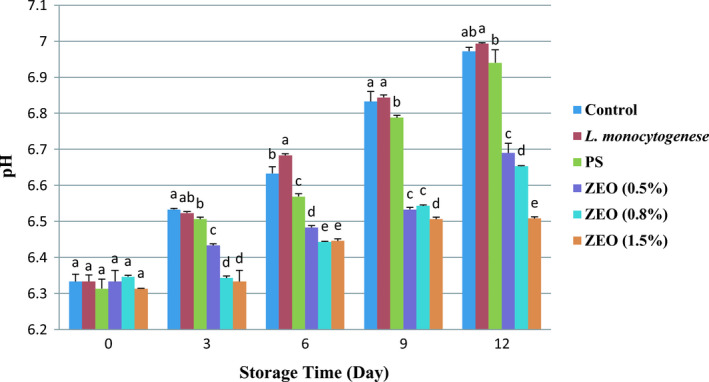
Changes (mean ± *SD*) of pH in rainbow trout fillets at different during 12 days storage at 4°C. Different letters indicate significant differences between days of storage

Different percentages of ZEO and storage day had a significant impact on pH during the experimental period (*p* <.05), (*p* <.001). Also, the interaction between the two factors was significant (*p* =.001). The lowest pH was recorded in 1.5% ZEO treatment from 6.31 ± 0.001 to 6.5 ± 0.004 during storage time. Figure 6 shows the increasing trend of pH. These outcomes confirm with Ozogul et al., (2017) study, effects of herb essential oils on quality of rainbow trout fillets during ice storage, which reported an upward trend in pH level during 24 days. Bacterial activity produces metabolic substances thus increasing the alkalinity of the environment (Khedri & Roomiani, 2019). Contrary to these results, Shadman et al., (2017) reported a significant decrease in the pH values.

### 3.8 Sensory evaluation

Table 2 shows the results of odor, color, taste, texture, and overall acceptability evaluations of rainbow fillets. In general, samples with ZEO received higher sensory evaluation scores than the control group and PS treatments. According to the overall acceptability index, the best score was observed for the fillets that had 0.8% and 1.5% of ZEO. These results agreed with Mousavi et al., (2019), who reported that sausage with 1.5% of ZEO had the highest overall acceptance score during refrigerated storage. Moreover, Khedri and Roomiani (2019) reported that silver carp fillets with 1.5% of ZEO had the highest sensory properties score during 12 days of storage. Another previous study by Jannatiha et al., (2020) on chicken legs showed that carboxymethyl cellulose film incorporating 2% ZEO decreased overall acceptability slightly. None of the samples could receive a score 6 in the current study during 12 days of storage. This study also showed the quality factors of rainbow trout fillets on day 9 were unacceptable. In the control group, it happened on day 6 of the storage time. In addition, the activity of microorganisms and enzymatic autolysis can reduce the shelf life and quality of fish meat. According to this study results, adding ZEO to rainbow trout fillets can keep the sensory property at an acceptable level for up to 6 days at refrigerator temperature.

**TABLE 2:** Sensory evaluations of rainbow trout fillets containing different concentrations of *Z. multiflora* essential oil (ZEO) during 12 days storage at 4 ± 1°C (mean ± *SD*)

Parameter	Treatment	Storage time (Day)				
		0	3	6	9	12
Odor	Control	8.1 ± 0.4^d^	7.1 ± 0.04^e^	6 ± 0.06^e^	5 ± 0.01^d^	2.5 ± 0.01^d^
	PS	8 ± 0.4^e^	7.8 ± 0.01^a^	6.1 ± 0.08^d^	5 ± 0.03^d^	2.4 ± 0.01^e^
	ZEO 0.5%	8.2 ± 0.01^c^	7.3 ± 0.01^d^	6.3 ± 0^c^	5.2 ± 0.08^c^	3.3 ± 0.01^c^
	ZEO 0.8%	8.4 ± 0.01^b^	7.4 ± 0.04^c^	6.5 ± 0.01^b^	5.5 ± 0.01^b^	4.5 ± 0.01^b^
	ZEO 1.5%	8.5 ± 0.02^a^	7.5 ± 0.02^b^	6.7 ± 0^a^	5.8 ± 0.02^a^	4.7 ± 0.01^a^
Color	Control	8.5 ± 0.03^c^	7.4 ± 0.03^b^	6.3 ± 0.02^c^	4.2 ± 0.03^e^	2.1 ± 0.03^d^
	PS	8.4 ± 0^d^	7.3 ± 0.01^c^	6.2 ± 0.03^d^	4.3 ± 0.03^d^	2 ± 0.03^e^
	ZEO 0.5%	8.5 ± 0.01^c^	7.2 ± 0.01^d^	6.3 ± 0.03^c^	5.2 ± 0.03^c^	3.1 ± 0.03^c^
	ZEO 0.8%	8.6 ± 0.01^b^	7.5 ± 0.03^a^	6.6 ± 0.03^b^	5.5 ± 0.02^b^	4.1 ± 0.01^b^
	ZEO 1.5%	8.7 ± 0^a^	7.4 ± 0.01^b^	6.7 ± 0.01^a^	5.7 ± 0.03^a^	4.3 ± 0.02^a^
Taste	Control	8.2 ± 0.02^d^	6.3 ± 0.01^e^	5.2 ± 0.03^e^	4.8 ± 0.03^e^	2.5 ± 0.03^c^
	PS	8 ± 0.03^e^	6.5 ± 0.02^d^	6.1 ± 0.02^d^	4.9 ± 0.03^d^	2.3 ± 0.03^d^
	ZEO 0.5%	8.3 ± 0.02^c^	7.4 ± 0.01^c^	6.8 ± 0.02^c^	5 ± 0.03^c^	3.2 ± 0.03^b^
	ZEO 0.8%	8.5 ± 0.03^a^	7.8 ± 0.02^a^	7.1 ± 0.02^a^	5.5 ± 0.03^a^	4.1 ± 0.02^a^
	ZEO 1.5%	8.4 ± 0.03^b^	7.6 ± 0.01^b^	6.9 ± 0.03^b^	5.4 ± 0.02^b^	4.1 ± 0.03^a^
Texture	Control	8.8 ± 0.02^b^	7.7 ± 0.03^c^	6.3 ± 0.02^e^	5.2 ± 0.02^d^	4.3 ± 0.01^c^
	PS	8.7 ± 0.02^c^	7.6 ± 0.01^d^	6.4 ± 0.02^d^	5 ± 0.01^e^	4.2 ± 0.02^d^
	ZEO 0.5%	8.9 ± 0.01^a^	7.7 ± 0.02^c^	6.5 ± 0.01^c^	5.5 ± 0.01^c^	4.3 ± 0.03^c^
	ZEO 0.8%	8.9 ± 0.03^a^	7.8 ± 0.03^b^	6.7 ± 0.03^b^	5.6 ± 0.02^b^	4.4 ± 0.03^b^
	ZEO 1.5%	8.8 ± 0.03^b^	7.9 ± 0.03^a^	6.8 ± 0.01^a^	5.8 ± 0.02^a^	4.5 ± 0.03^a^
Overall	Control	8.2 ± 0.02^c^	6.7 ± 0.02^d^	5.2 ± 0.02^e^	4.1 ± 0.03^e^	3.2 ± 0.01^d^
	PS	8.3 ± 0.03^d^	6.6 ± 0.03^e^	6.1 ± 0.03^d^	5.5 ± 0.01^d^	4.1 ± 0.01^c^
	ZEO 0.5%	8.2 ± 0.02^c^	7.1 ± 0.02^c^	6.3 ± 0.03^c^	5.7 ± 0.03^c^	4.3 ± 0.03^b^
	ZEO 0.8%	8.6 ± 0.03^a^	7.5 ± 0^a^	6.6 ± 0.03^a^	5.8 ± 0.03^b^	4.5 ± 0.02^a^
	ZEO 1.5%	8.5 ± 0.03^b^	7.4 ± 0.03^b^	6.5 ± 0.02^b^	5.9 ± 0.02^a^	4.5 ± 0.02^a^

## 4 CONCLUSIONS

In this study, rainbow trout was selected as the substrate to investigate the antibacterial and antioxidant properties of ZEO against *L. monocytogenes*. The use of 1.5% ZEO on rainbow trout fillets had the best effect on the reduction growth of inoculated *L. monocytogenes*, TVC as well as TVB‐N, TBA, and levels of *P. aeruginosa*. As a result, it extended the shelf life of raw rainbow trout fillets. Therefore, natural preservatives can be replaced with chemicals. Hence, considering the acceptable antibacterial effect and sensorial scores of ZEO, it could practically use in the food industry as a natural antimicrobial and antioxidant agent, especially in raw fish fillets.

## CONFLICT OF INTEREST

The authors declare that they have no conflict of interest.
